# Comparing the effectiveness of Chinese patent medicines containing red yeast rice on hyperlipidaemia: A network meta‐analysis of randomized controlled trials

**DOI:** 10.1002/edm2.314

**Published:** 2021-11-11

**Authors:** Guiqin Xu, Mingxin Lin, Xueli Dai, Jingqing Hu

**Affiliations:** ^1^ Affiliated Hospital of Integrated Traditional Chinese and Western Medicine Nanjing University of Chinese Medicine Nanjing China; ^2^ Institute of Basic Theory for Chinese Medicine China Academy of Chinese Medical Sciences Beijing China; ^3^ Department of Scientific Research Management The First Affiliated Hospital of Anhui University of Chinese Medicine Hefei China; ^4^ The First Affiliated Hospital Anhui University of Chinese Medicine Hefei China

**Keywords:** Chinese patent medicine, evidence‐based medicine, network meta‐analysis

## Abstract

**Introduction:**

The purpose of this study was to evaluate the therapeutic effectiveness of Chinese patent medicines containing red yeast rice for the treatment of hyperlipidaemia.

**Methods:**

Relevant literature published until 13 August 2021, was retrieved from six electronic databases. Randomized clinical trials of Chinese patent medicines containing red yeast rice in patients with hyperlipidaemia were included in the review. Network meta‐analysis was performed using Stata 13.1 software. Methodological quality was assessed using the Cochrane risk of bias tool. The surface under the cumulative ranking (SUCRA) curve probability values were used to rank the treatments.

**Results:**

This study included 47 trials involving 4824 subjects. In terms of reduced total cholesterol levels, Xuezhikang (SUCRA: 84.5%) had the highest probability of being the most effective formulation, with Simvastatin (66.4%) and Zhibitai (65.4%) ranked second and third, respectively. Xuezhikang also had the highest probability of reducing low‐density lipoprotein cholesterol levels to the greatest extent (SUCRA: 82.6%) with Simvastatin (SUCRA: 74.9%) and Zhibituo (SUCRA: 52.8%) being the second and third choices, respectively. For reduced triglyceride levels, Zhibituo (SUCRA: 80.2%) exhibited the highest probability of being the most effective, with Xuezhikang (SUCRA: 63.4%) and Simvastatin (SUCRA: 57.6%) in second and third places, respectively. Finally, in terms of improving high‐density lipoprotein cholesterol levels, Zhibituo (SUCRA: 90.1%) had the highest probability of being the most effective, with Simvastatin (SUCRA: 82.1%) and Xuezhikang (SUCRA: 51.1%) ranked second and third, respectively.

**Conclusions:**

Xuezhikang was found to have the highest probability of being the most effective formulation for reducing total cholesterol and low‐density lipoprotein cholesterol levels, while Zhibituo had the highest probability of being the most effective for controlling triglyceride and high‐density lipoprotein cholesterol levels. The studies included in the review exhibited certain limitations and, therefore, more rigorously designed studies should be performed.

Trial registration: INPLASY registration number: INPLASY202130017.

## Graphical abstract

The purpose of the present study was to evaluate the therapeutic effectiveness of Chinese patent medicines containing red yeast rice for the treatment of hyperlipidaemia. Xuezhikang was found to have the highest probability of being the most effective formulation for reducing total cholesterol and low‐density lipoprotein cholesterol levels, while Zhibituo had the highest probability of being most effective for controlling triglyceride levels and high‐density lipoprotein cholesterol levels. The studies included in the review exhibited a number of limitations, and therefore, more rigorously designed studies should be performed.

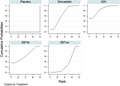

## 1 INTRODUCTION

Hyperlipidaemia is a common, global metabolic syndrome associated with lipid abnormalities, including increased levels of triglycerides (TG), total cholesterol (TC) and low‐density lipoprotein cholesterol (LDL‐C) and decreased levels of high‐density lipoprotein cholesterol (HDL‐C).^1^, ^2^ Serum lipid levels in the Chinese population have gradually increased, and the prevalence of dyslipidaemia among Chinese adults has reached 40.4%.^3^, ^4^, ^5^ Hyperlipidaemia is a major contributory factor to various diseases, including cardiovascular diseases, type 2 diabetes, Alzheimer's disease and Parkinson's disease.^6^, ^7^, ^8^ Therefore, measures to effectively and treat dyslipidaemia are crucial for preventing cardiovascular and cerebrovascular diseases.

Statins are currently the drug of choice for the treatment of hypercholesterolemia.^9^, ^10^ However, the side effects caused by their use often limit their application.^11^ Previous studies have suggested that extracts of red yeast rice (RYR) reduce blood lipid levels.^12^, ^13^ There are many varieties of oral Chinese patent medicines containing RYR for the treatment of hyperlipidaemia, such as Xuezhikang, Zhibituo and Zhibitai capsules, which are widely used in China for the treatment of hyperlipidaemia.^14^

However, the efficacy of these Chinese patent medicines has not been directly compared for the treatment of hyperlipidaemia; therefore, it is not possible to select an optimal formulation for patients with hyperlipidaemia. Consequently, we conducted a network meta‐analysis to compare the therapeutic effectiveness of Chinese patent medicines for treating hyperlipidaemia and identify which of them was consistently ranked as the most effective.

## 2 MATERIALS AND METHODS

The protocol for this meta‐analysis was registered using the INPLASY (No. INPLASY202130017), available on Inplasy.com (https://doi.org/10.37766/inplasy2021.3.0017). Ethics approval for this study was not required, as the meta‐analysis did not involve identifiable patient data.

### 2.1 Bibliographic search strategy

Two authors (XGQ and DXL) conducted the literature searches. Supplementary searches were performed using Google Scholar software. Searches were restricted to studies published in English or Chinese. A representative example of the search strategy using the PubMed database is as follows: #1 (hyperlipidemias[MeSH Terms]) OR (cholesterol[MeSH Terms]); #2 (random allocation[MeSH Terms]) OR (randomized)) OR (placebo) OR randomized controlled trial; #3 Simvastatin[MeSH Terms]; #4 ((Xuezhikang[Title/Abstract]) OR (Zhibituo[Title/Abstract])) OR (Zhibitai[Title/Abstract]); #5 #1 AND #2 AND #3 AND #4.

### 2.2 Inclusion criteria

Trials were included in the present study following the PICOS framework (population, intervention, comparisons, outcomes and study type). Population (P): participants were diagnosed with hyperlipidaemia according to recognized diagnostic criteria, with no limitations in terms of age, gender, or ethnicity. Intervention (I): The experimental group was prescribed any of the following Chinese patent medicines: Xuezhikang capsules, Zhibituo capsules or Zhibitai capsules, without the co‐administration of Western medicine. Comparisons (C): The control group received Simvastatin (Zocor) or placebo, and pairwise comparisons of the above Chinese patent medicines were performed. Outcomes (O) were serum lipid levels, including TC, TG, LDL‐C, HDL‐C levels and adverse drug reactions (ADRs). Study type (S): Only randomized controlled trials (RCTs) were included, and trials in languages other than Chinese or English were excluded.

### 2.3 Exclusion criteria

Studies were excluded for the following reasons: (1) duplicate publications; (2) case reports, reviews, or studies with animals as research subjects; (3) patient comorbidities (eg diabetes, cardiovascular diseases and cerebrovascular diseases), (4) incorrect or missing data or (5) trials with <50 cases.

### 2.4 Outcome measures

The main outcomes were serum lipid levels, including TC, TG, LDL‐C and HDL‐C levels. The secondary outcomes included ADRs.

### 2.5 Data extraction

Two reviewers (XGQ and DXL) independently selected the studies. Titles and abstracts were screened to identify potential articles, and then, the full texts of the screened articles were read to determine suitable studies based on the inclusion and exclusion criteria. Discrepancies in selection were resolved through team discussion. The selection procedures were based on the Preferred Reporting Items for Systematic Reviews and Meta‐Analyses (PRISMA) flow chart.^15^

### 2.6 Assessment of quality

Two researchers (XGQ and DXL) independently assessed the risk of bias in the studies included in this review using the risk of bias tool from the Cochrane Handbook. Any disagreements were resolved using an arbiter. The following items were evaluated: selection bias (random sequence generation, allocation concealment), performance bias (blinding of participants and personnel), detection bias (blinding of assessors), attrition bias and other types of bias. The Grades of Recommendation, Assessment, Development and Evaluation (GRADE) approach was used to assess the quality of evidence.^16^ According to the GRADE approach, evidence quality is classified into four levels: high, moderate, low and very low. RCTs provide high‐quality evidence; however, the evidence can be downgraded from high to low quality owing to five factors: study limitation (risk of bias), indirectness, inconsistency, imprecision and publication bias.

### 2.7 Statistical analysis

A statistical software (Stata 13.1; Stata Corporation) was used for the present study. The results were reported as mean differences (MDs) with 95% confidence intervals (CIs). Heterogeneity assessment was performed using chi‐squared (*χ* ^2^) tests; if *I* ^2^ was ≤50%, the heterogeneity was considered to be low, and network meta‐analysis could be performed; if *I* ^2^ > 50%, heterogeneity was deemed to be high, and the study could be conducted when the source of heterogeneity could be found. For indirect comparisons, the treatment effects of all regimens were estimated using a two‐stage network meta‐analysis as follows: Initially, an inconsistency test was performed using a node‐splitting model, and fitting consistency or inconsistency models were constructed and presented using the network command; if inconsistency was not statistically significant (*p* > .05), a consistency model was used; otherwise, an inconsistency model was employed. Pairwise comparisons were conducted using the ‘interval plot’ command. Ranking probabilities for each intervention were then estimated using the ‘network rank’ command. Surface under the cumulative ranking (SUCRA) curve values were calculated to rank the efficacy of each intervention. Larger SUCRA values indicate a more effective intervention. Publication bias was evaluated using comparison‐adjusted funnel plots.

## 3 RESULTS

### 3.1 Descriptions of studies

**FIGURE 1:**
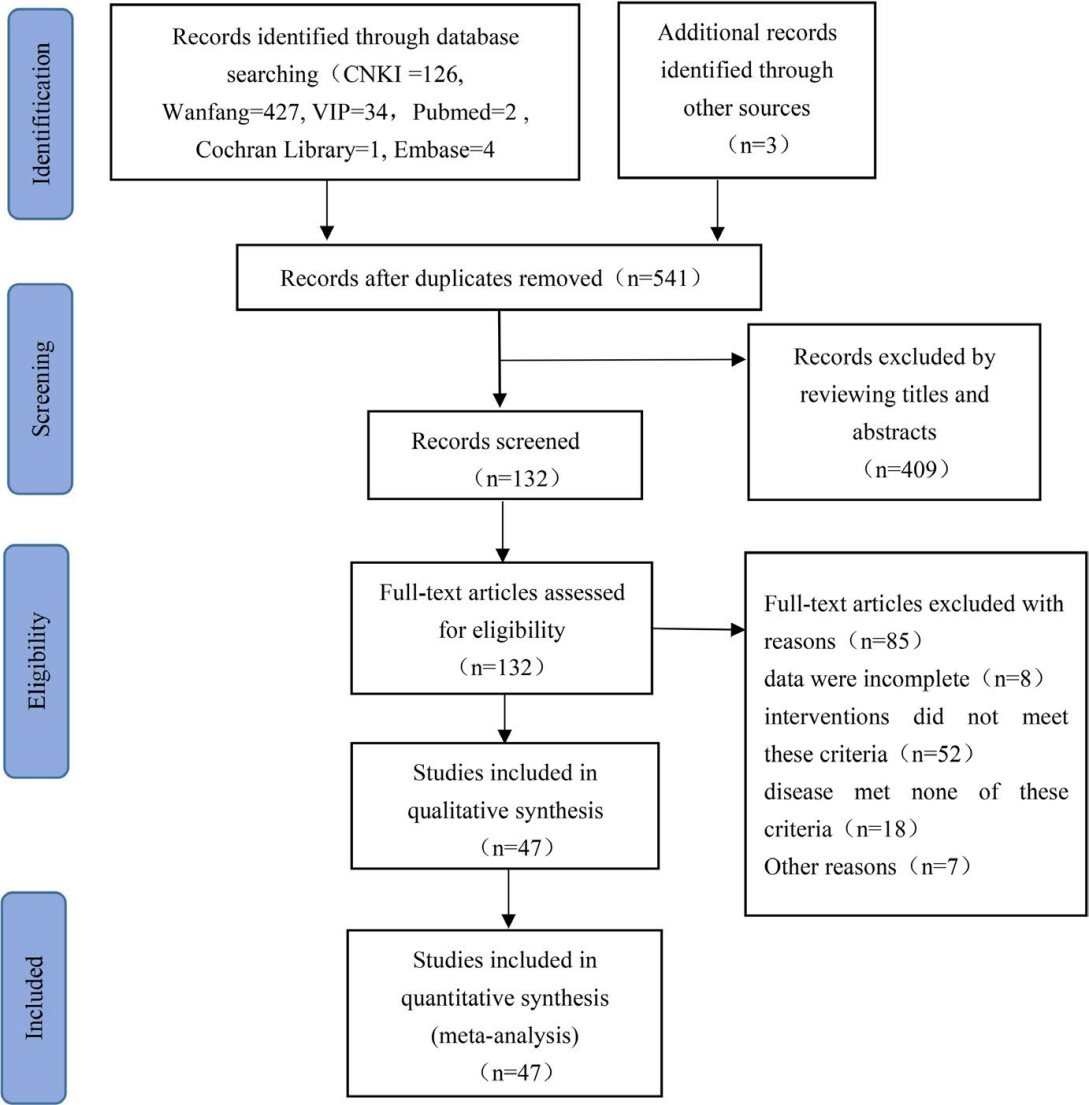
Flow chart of selection of studies

A total of 597 relevant studies were initially selected, of which 53 duplicates were excluded. Another 409 articles were excluded after reviewing their titles and abstracts based on the inclusion and exclusion criteria. Finally, the full text of the remaining 132 articles was read, from which 47 RCTs were identified as satisfying the inclusion criteria and were included in the final analysis. A flowchart describing the retrieval and screening processes is shown in Figure 1.

### 3.2 Baseline characteristics of included studies

A total of 47 RCTs involving 4824 participants diagnosed with hyperlipidaemia satisfied the study selection criteria and were included in the study. From these 47 RCTs, the effects on hyperlipidaemia resulting from the use of three Chinese patent medicines containing RYR were summarized. The characteristics of the included studies are summarized in Table 1. The included studies showed that all baseline values were comparable.

**TABLE 1:** Characteristics of included studies in the network meta‐analysis

No.	Study	Year	No. of patients	Average age (T/C)	Treatment 1 drug dose		Treatment 2 drug dose		Duration	Outcome measures
1	He Y^17^	2013	88	48.2 ± 13.5/47.2 ± 14.4	ZBTai	240 mg, Bid	Simvastatin	10 mg, QD	8 weeks	①②③④⑤
2	Wu GZ^18^	2010	192	60.5	ZBTuo	300 mg, Tid	Simvastatin	10 mg, QD	12 weeks	②③④⑤⑥
3	Hu XZ^19^	2011	128	61.2 ± 3.5	ZBTuo	300 mg, Tid	Simvastatin	10 mg, QD	3 months	②③④⑤⑥
4	Zhao S^20^	2008	99	51.2/51.5	XZK	600 mg, QD	Simvastatin	10 mg, QD	4 weeks	②③④⑤⑥
5	Li ZH^21^	2017	92	74.47 ± 5.38/62.34 ± 2.72	XZK	600 mg, Bid	Simvastatin	20 mg, QD	8 weeks	②③④⑤ hs‐CRP
6	Xue SL^22^	2010	108	50.34 ± 10.28/51.61 ± 10.09	XZK	600 mg, Bid	Simvastatin	20 mg, QD	24 weeks	②③④⑤⑥ hs‐CRP, IMT
7	Zhang G^23^	1998	80	57.67 ± 9.69/57.58 ± 8.74	XZK	600 mg, Bid	Simvastatin	10 mg, QD	4 weeks	①②③④⑤⑥
8	Xi BL^24^	2002	60	69.57 ± 6.99/70.60 ± 5.65	XZK	600 mg, Bid	Simvastatin	20 mg, QD	4 weeks	②③④⑤⑥
9	Chen QY^25^	2007	82	59.4 ± 3.2 /60.2 ± 4.2	XZK	600 mg, Bid	Simvastatin	20 mg, QD	4 weeks	②③④⑤⑥
10	Chen LL^26^	2002	65	55.2 ± 3.8/56.7 ± 3.1	XZK	1200 mg, QN	Simvastatin	10 mg, QD	4 weeks	①②③④⑤⑥
11	Zhang XF^27^	2010	76	55.6 ± 8.7/56.5 ± 9.1	XZK	1200 mg, Bid	Simvastatin	20 mg, QD	8 weeks	②③④⑤⑥
12	Liu SP^28^	2013	120	58	XZK	600 mg, Bid	Simvastatin	20 mg, QD	8 weeks	①②③④⑤⑥
13	Zhu QF^29^	2003	159	62.32 ± 12.27/61.53 ± 11.69	XZK	600 mg, Bid	Simvastatin	20 mg, QD	3 months	②③④⑤⑥⑦⑧
14	Zheng W^30^	2013	61	59.8 ± 9.7	XZK	600 mg, Bid	Simvastatin	20 mg, QD	12 weeks	①②③④⑤⑥
15	Li KL^31^	2006	80	55.53 ± 10.56/56.05 ± 10.22	XZK	600 mg, Bid	Simvastatin	20 mg, QD	8 weeks	②③④⑥
16	Qi MY^32^	2004	224	Not reported	XZK	1200 mg, Bid	Simvastatin	10 mg, QD	8 weeks	①②③④⑤⑥
17	Hua C^33^	2011	100	45–75	XZK	600 mg, Bid	Simvastatin	10 mg, QD	8 weeks	②④
18	Wang SH^34^	2003	120	69.57 ± 6.99/70.60 ± 5.65	XZK	600 mg, Bid	Simvastatin	20 mg, QD	4 weeks	②③④⑤⑥
19	Chen FJ^35^	2003	286	60.17 ± 8.52	XZK	600 mg, Bid	Simvastatin	10 mg, QD	12 weeks	②③④⑤⑥⑦⑧
20	Zhou H^36^	2012	139	59.13 ± 9.20/61.42 ± 8.52	ZBTai	240 mg, Bid	Simvastatin	20 mg, QD	12 months	②③④⑤⑥⑨
21	Pang J^37^	2018	60	65/63.5	ZBTai	240 mg, Bid	Simvastatin	20 mg, QD	4 weeks	②③④
22	Yang WJ^38^	2003	70	64.03 ± 5.71/63.07 ± 6.20	ZBTuo	240 mg, Bid	Simvastatin	20 mg, QD	8 weeks	①②③④⑤⑥
23	Liu JX^39^	2005	68	58 ± 12/56 ± 11	ZBTuo	240 mg, Bid	Simvastatin	20 mg, QD	8 weeks	①②③④⑤⑥
24	Guo XM^40^	1999	63	56 ± 12/58 ± 11	ZBTuo	480 mg, Bid	Simvastatin	10 mg, QD	6 weeks	①②③④⑤⑥⑦⑧
25	Zhang GR^41^	2002	137	59.25/58.85	ZBTuo	1050 mg, Tid	Simvastatin	10 mg, QD	4 weeks	①②③④⑤⑥
26	Peng KL^42^	2011	220	Not reported	ZBTuo	300 mg, Tid	Simvastatin	10 mg, QD	4 months	②③④⑤⑥
27	Xu J^43^	2009	100	56.2/56.9	ZBTuo	1050 mg, Tid	Simvastatin	20 mg, QD	8 weeks	①②③④⑤⑥
28	Zhang QL^44^	2004	60	56.5/58	ZBTuo	1050 mg, Tid	Simvastatin	20 mg, QD	8 weeks	①②③④⑤⑥
29	Feng ZH^45^	2006	100	57.9/56.8	ZBTuo	1050 mg, Tid	Simvastatin	20 mg, QD	8 weeks	①②③④⑤⑥
30	Guo SH^46^	2019	100	55.3 ± 3.2/54.2 ± 3.5	XZK	600 mg, Bid	Simvastatin	20 mg, QD	12 weeks	①②③④⑤⑥
31	Li XL^47^	2011	68	58 ± 12/56 ± 11	ZBTuo	240 mg, Bid	Simvastatin	20 mg, QD	8 weeks	②③④⑤⑥
32	Zhao PF^48^	2011	120	67.32 ± 9.42/65.93 ± 8.83	ZBTuo	1050 mg, Bid	Simvastatin	20 mg, QD	8 weeks	②③④⑤⑥
33	Zhang Q^49^	2015	96	62.6 ± 7.8/60.8 ± 8.3	XZK	600 mg, Bid	Simvastatin	20 mg, QD	8 weeks	①②③④⑤, ⑥, CRP, Hcy, Cost‐effect analysis
34	Duan CM^50^	2014	70	41.9 ± 3.7/42.8 ± 3.1	XZK	600 mg, Bid	Placebo	600 mg, Bid	16 weeks	①②③④⑤⑥
35	Yang WX^51^	2013	84	49 ± 8.7/49 ± 9.1	XZK	600 mg, Bid	Simvastatin	20 mg, QD	8 weeks	①②③④⑤
36	Lu XB^52^	2012	60	50.75 ± 3.72/43.33 ± 6.03	XZK	600 mg, Bid	Placebo	600 mg, Bid	8 weeks	①②③④⑤⑥⑩
37	Xu NF^53^	2011	60	51.84 ± 10.16/51.36 ± 10.65	XZK	600 mg, Bid	Placebo	600 mg, Bid	8 weeks	①②③④⑤⑥⑩
38	Chen L^54^	2010	100	56.5 ± 7.1	Simvastatin	20 mg, QD	XZK	600 mg, Bid	8 weeks	②③④⑤⑥
39	Wang M^55^	2005	160	56.3 ± 9.1/57.4 ± 10.2	ZBTuo	1050 mg, Bid	Simvastatin	20 mg, QD	8 weeks	①②③④⑤⑥
40	Chen L^56^	2003	117	56 ± 15/53 ± 18	ZBTuo	1050 mg, Bid	XZK	600 mg, Bid	30 days	②③④⑤⑥
41	Chen ZM^57^	2001	90	54 ± 9/56 ± 9	Simvastatin	10 mg, QD	ZBTuo	1050 mg, Tid	8 weeks	②③④⑤⑥
42	Chen SM^58^	2001	58	57.8/56.4	XZK	600 mg, Bid	Placebo	600 mg, Bid	8 weeks	②③④⑤, internal diameter of the brachial artery
43	Lu YS^59^	1997	62	53 ± 8/53 ± 6	XZK	600 mg, Bid	ZBTuo	1050 mg, Tid	8 weeks	②③④⑤
44	Zhao DY^60^	2012	60	52.6 ± 9.34/55.46 ± 11.70	XZK	600 mg, Bid	Placebo	600 mg, Bid	8 weeks	②③④⑤⑥, improvement of clinical symptoms
45	Yu JB^61^	2014	60	18–75	XZK	600 mg, Bid	Placebo	600 mg, Bid	8 weeks	②③④⑤⑥
46	Qi RY^62^	2007	162	65.5 ± 4.32/67.4 ± 5.2	XZK	600 mg, Bid	Placebo	600 mg, Bid	8 weeks	①②③④⑤⑥
47	Peng DY^63^	1998	60	68 ± 3/66 ± 5	ZBTuo	1050 mg, Tid	Placebo	1050 mg, Tid	6 weeks	①②③⑤⑥

Abbreviations: ①, clinical efficacy; ②TC, total cholesterol; ③TG, triglycerides; ④LDL‐C, low‐density lipoprotein cholesterol; ⑤HDL‐C, high‐density lipoprotein cholesterol; ⑥ADR, adverse drug reaction; ⑦Apolipoprotein A1;⑧Apolipoprotein B;⑨Liver and kidney function, creatine kinase, carotid intima thickness;⑩Chinese medicine syndrome; C, control group; T, experimental group; XZK, Xuezhikang capsules; ZBTai, Zhibitai capsules; ZBTuo, Zhibituo capsules or Zhibituo tablets.

### 3.3 Risk of bias in included studies

**FIGURE 2:**
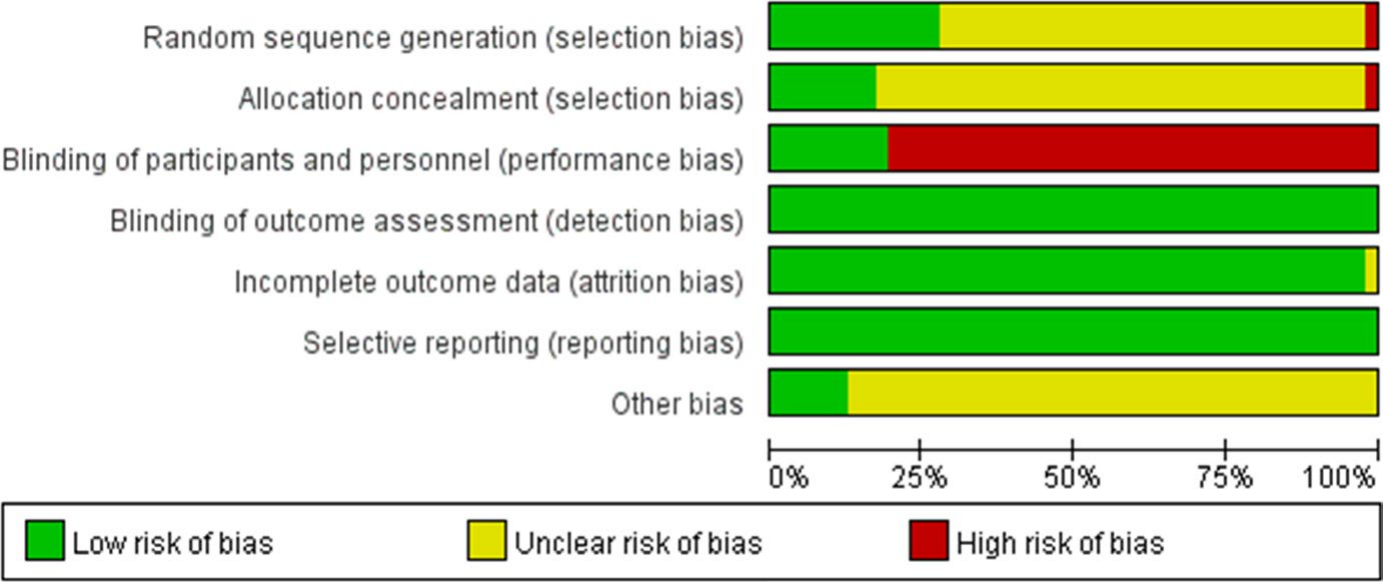
Risk of bias in the selected trials

The risk‐of‐bias graphs for the 47 studies are shown in Figure 2. All studies included randomization, five^21^, ^22^, ^28^, ^37^, ^46^ described the generation of a random sequence using a random number table, three^36^, ^52^, ^53^ were randomized, double‐blind, controlled trials, and four^50^, ^58^, ^61^, ^63^ were randomized, placebo‐controlled trials. Other studies have not described a specific method for random sequence generation. None of the unblinded studies stated the details of allocation concealment. For the blinding of participants and personnel, a high risk was identified in the unblinded studies. Because the outcomes were tested in the lab, the risk of blinding on the outcome assessment was low. All studies reported complete outcome data and were free of selective reporting. The unblinded studies were unclear in terms of other biases.

### 3.4 Outcome measures

#### 3.4.1 Test of inconsistency and network plot

**FIGURE 3:**
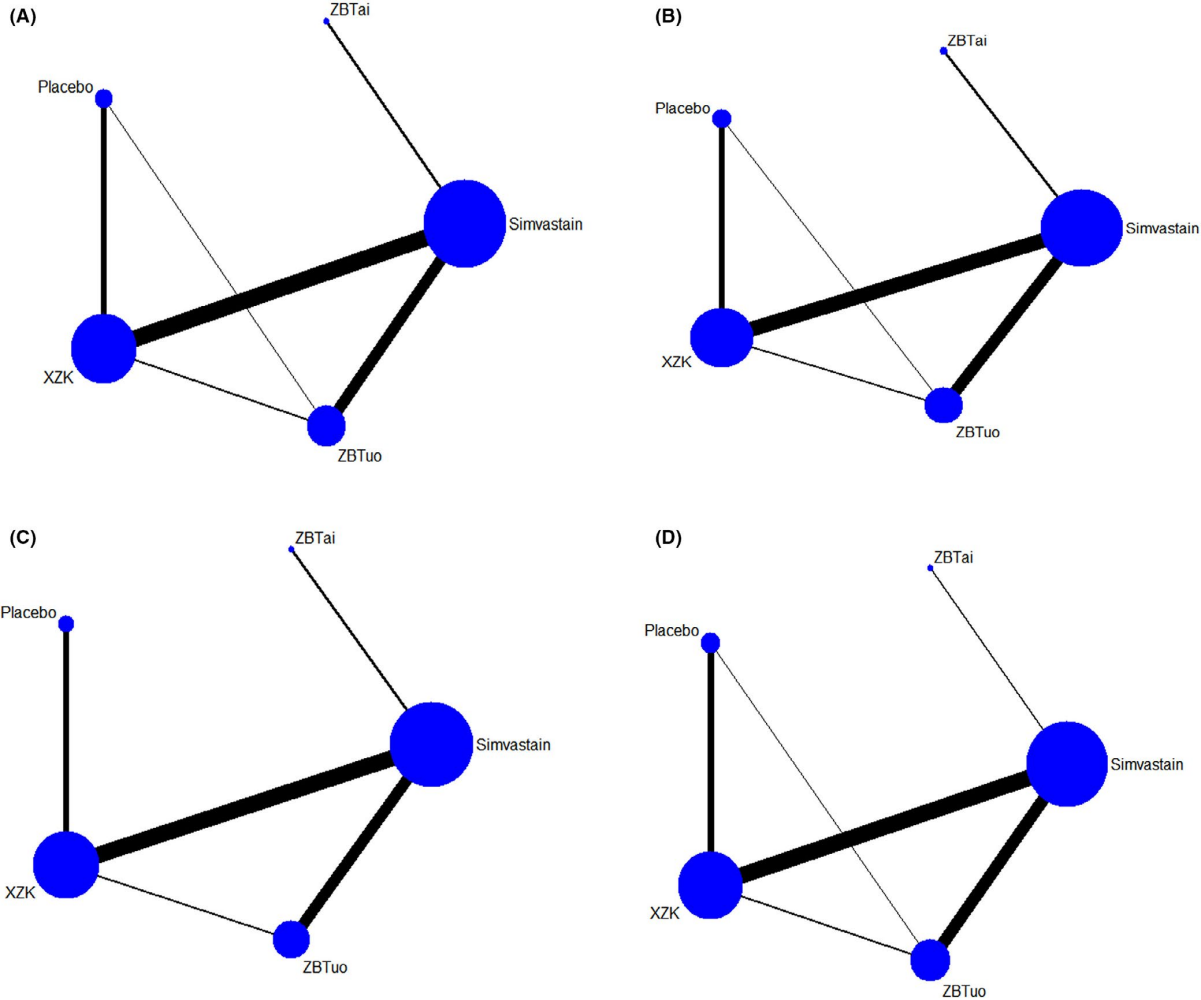
Network plot for TC (A), TG (B), LDL‐C (C) and HDL‐C (D). The nodes in the figure represent the following interventions XZK, Xuezhikang capsules; ZBTuo, Zhibituo capsules

The evidence network is shown in Figure 3. The network meta‐analysis included closed loops, and formal tests for inconsistency were performed. We found inconsistencies were not statistically significant (TC, *p* = .58; TG, *p* = .26; LDL‐C, *p* = .64; and HDL‐C, *p* = .32), and a network meta‐analysis was performed using a consistency model.

#### 3.4.2 TC—Total cholesterol levels

**FIGURE 4:**
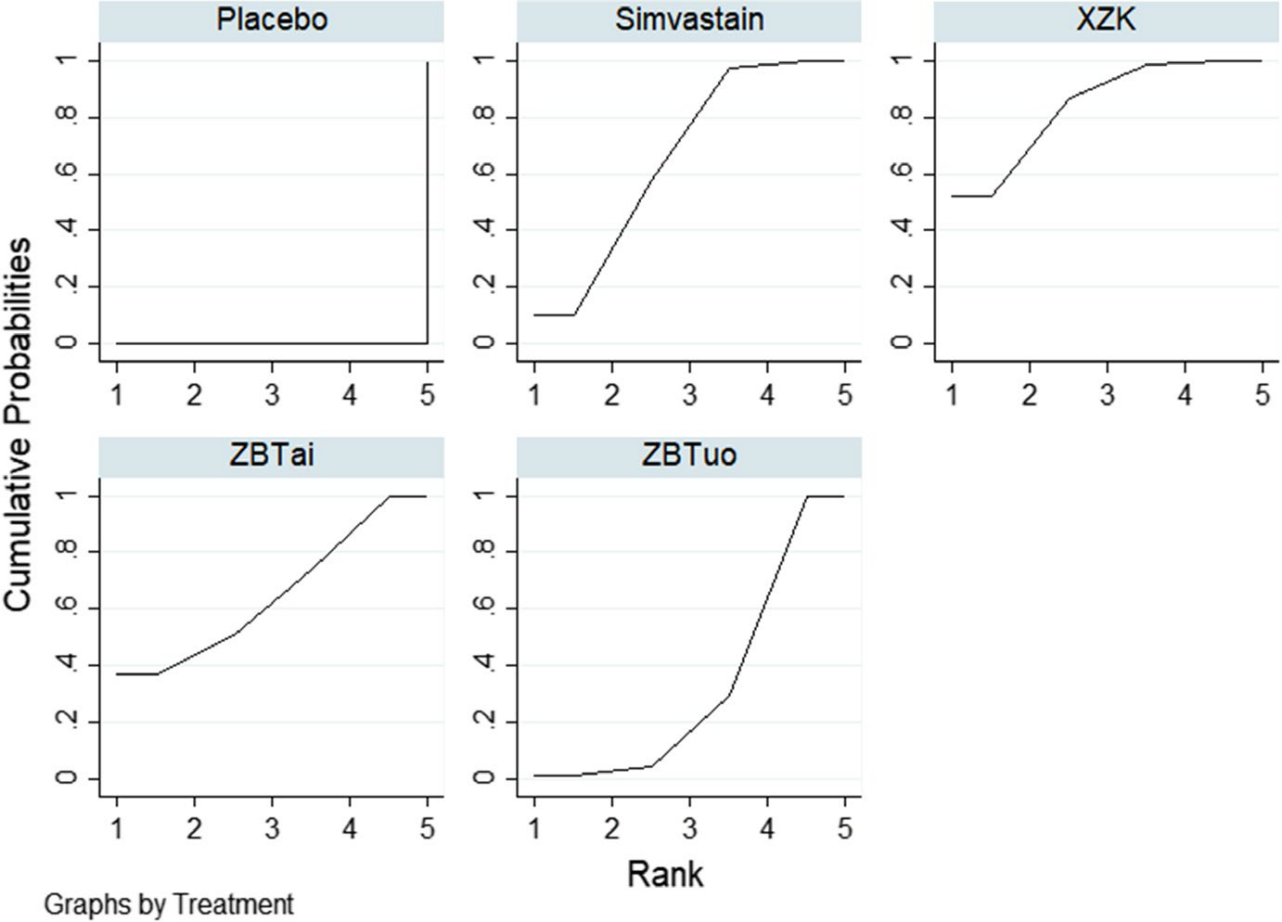
SUCRA curves for TC

For a total of 4803 patients in 47 RCTs,^17^, ^18^, ^19^, ^20^, ^21^, ^22^, ^23^, ^24^, ^25^, ^26^, ^27^, ^28^, ^29^, ^30^, ^31^, ^32^, ^33^, ^34^, ^35^, ^36^, ^37^, ^38^, ^39^, ^40^, ^41^, ^42^, ^43^, ^44^, ^45^, ^46^, ^47^, ^48^, ^49^, ^50^, ^51^, ^52^, ^53^, ^54^, ^55^, ^56^, ^57^, ^58^, ^59^, ^60^, ^61^, ^62^, ^63^ changes in TC levels after administration of three types of Chinese patent medicines were compared with changes due to Simvastatin or placebo. Chi‐squared tests showed no heterogeneity between studies (*p* = 1.0, *I* ^2^ = 0.0%). There was a statistically significant difference in all treatments compared with the placebo group. No statistically significant differences were observed between the other comparisons. The results of the network meta‐analysis are summarized in Table 2. The intervention ranking probabilities based on SUCRA were as follows: Xuezhikang (84.5%) > Simvastatin (66.4%) > Zhibitai (65.4%) > Zhibituo (33.7%) > placebo (0.0%) (Figure 4), suggesting that Xuezhikang was the most effective intervention for TC, with Simvastatin and Zhibitai ranking second and third, respectively.

**TABLE 2:** Network meta‐analysis for TC (MD [95% CI])

Zhibituo				
0.17 (−0.35, 0.70)	Zhibitai			
0.25 (−0.01, 0.50)	0.07 (−0.44, 0.59)	Xuezhikang		
0.17 (−0.03, 0.38)	0.00 (−0.48, 0.48)	−0.07 (−0.25, 0.10)	Simvastatin	
**−0.87 (−1.24**, **−0.49)**	**−1.04 (−1.63**, **−0.45)**	**−1.11 (−1.42**, **−0.81)**	**1.04 (−1.38**, **−0.69)**	Placebo

Bold values indicate statistically significant values *p* <  0.05.

#### 3.4.3 TG—Triglyceride levels

**FIGURE 5:**
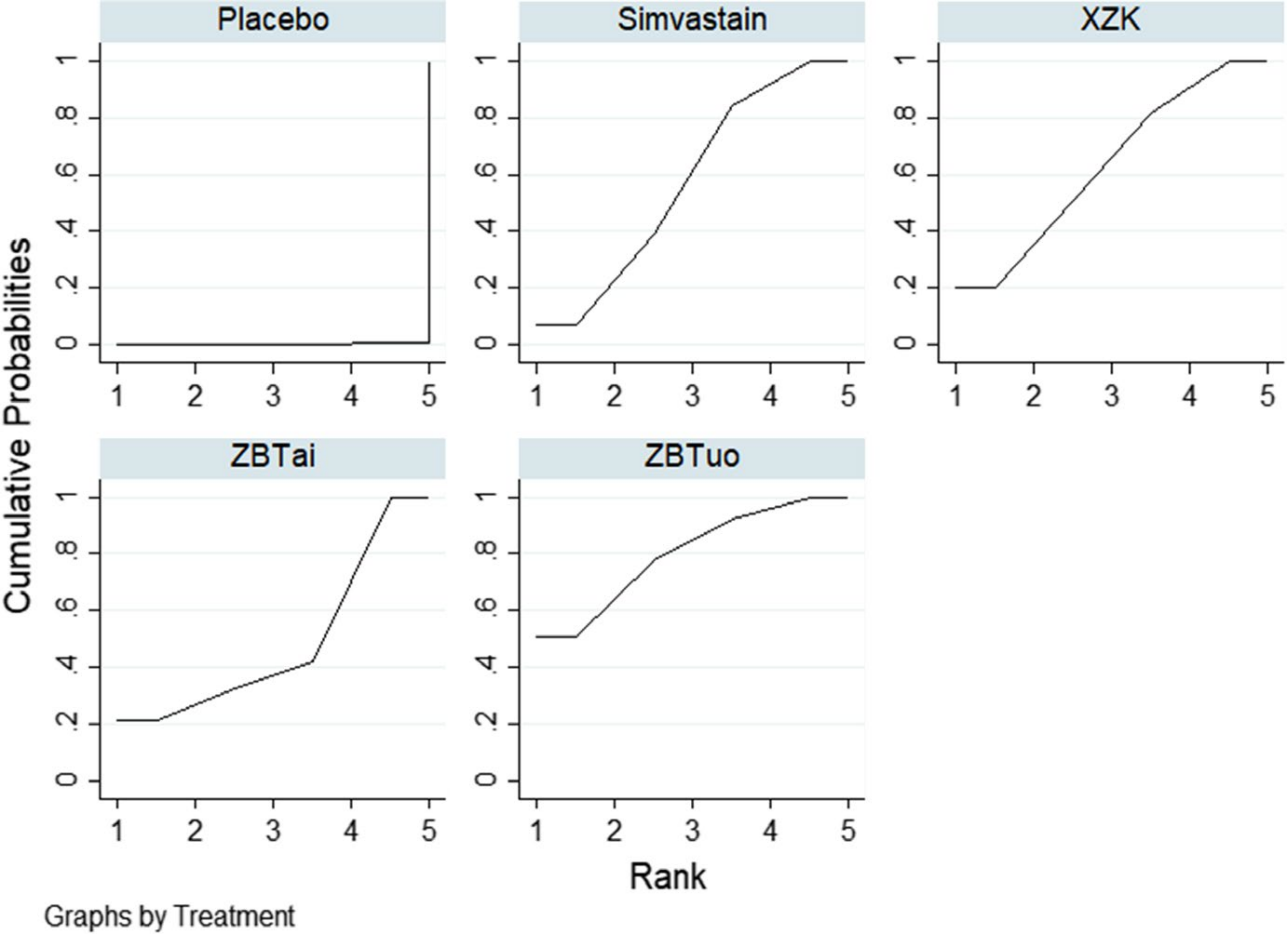
SUCRA curves for TG

A total of 4591 patients in 45 RCTs^17^, ^18^, ^19^, ^21^, ^22^, ^23^, ^24^, ^25^, ^26^, ^27^, ^28^, ^29^, ^30^, ^31^, ^32^, ^34^, ^35^, ^36^, ^37^, ^38^, ^39^, ^40^, ^41^, ^42^, ^43^, ^44^, ^45^, ^46^, ^47^, ^48^, ^49^, ^50^, ^51^, ^52^, ^53^, ^54^, ^55^, ^56^, ^57^, ^58^, ^59^, ^60^, ^61^, ^62^, ^63^ were treated with three types of Chinese patent medicines for TG, which were compared with Simvastatin or placebo. Heterogeneity tests showed no heterogeneity between studies (*p* = 1.0, *I* ^2^ = 0.0%). One study^20^ was excluded from the analysis because it contained an outlier value. There was a statistically significant difference in all treatments compared with the placebo group. No statistically significant differences were observed between the other comparisons. The results of the network meta‐analysis are summarized in Table 3. Based on the SUCRA values (Figure 5), Zhibituo (80.2%) > Xuezhikang (63.4%) > Simvastatin (57.6%) > Zhibitai (48.7%) > placebo (0.1%), suggesting that Zhibituo was the most effective intervention for TG, with Xuezhikang and Simvastatin ranking second and third, respectively.

**TABLE 3:** Network meta‐analysis for TG (MD [95% CI])

Zhibituo				
0.07 (−0.12, 0.26)	Zhibitai			
−0.08 (−0.50, 0.34)	−0.15 (−0.61, 0.31)	Xuezhikang		
0.01 (−0.15, 0.18)	−0.06 (−0.29, 0.18)	0.09 (−0.36, 0.55)	Simvastatin	
**−0.79 (−1.12**, **−0.46)**	**−0.86 (−1.22**, **−0.50)**	**−0.71 (−1.25**, **−0.17)**	**−0.80 (−1.10**, **−0.51)**	Placebo

Bold values indicate statistically significant values *p* <  0.05.

#### 3.4.4 LDL‐C—Low‐density lipoprotein cholesterol levels

**FIGURE 6:**
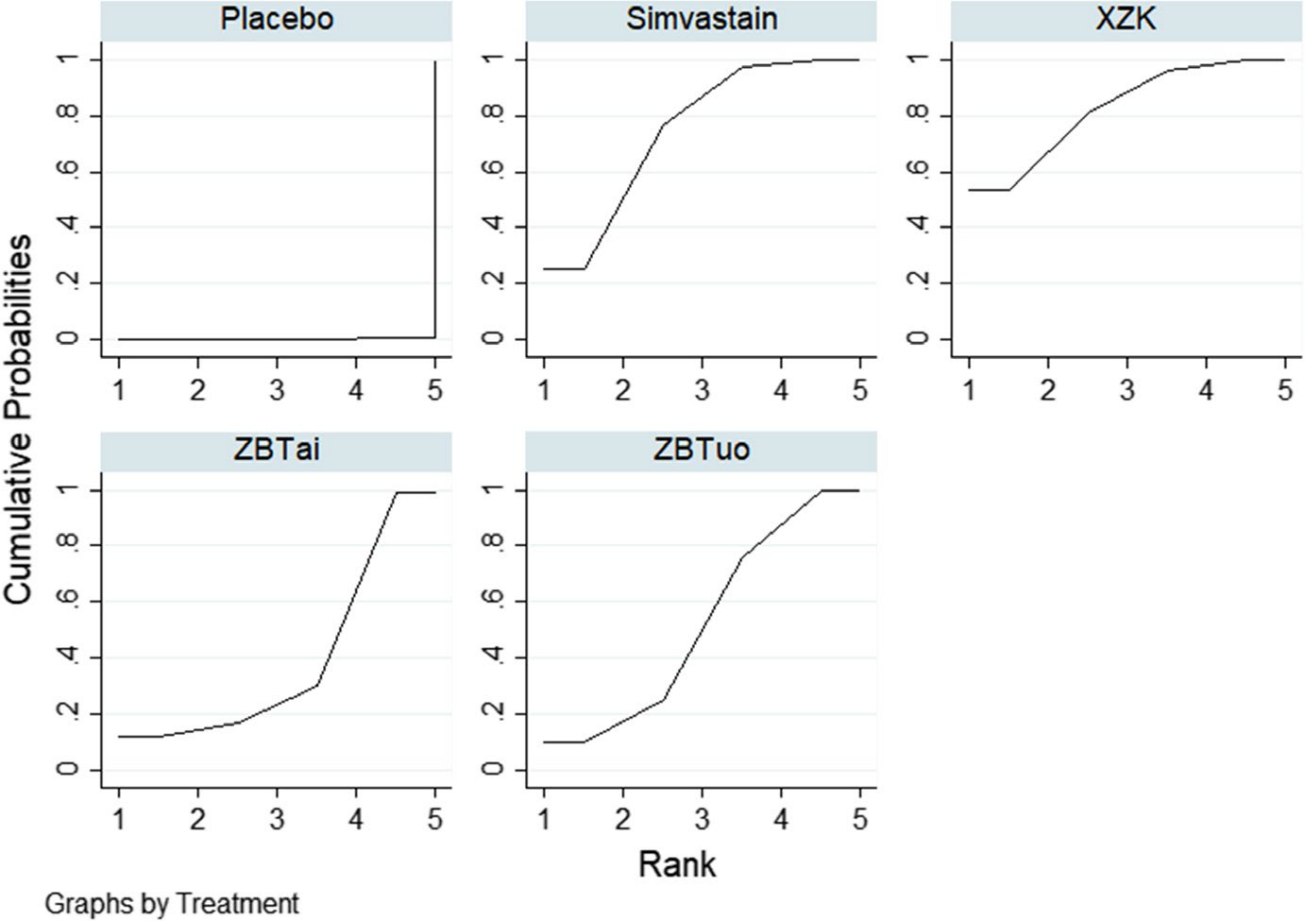
SUCRA curves for LDL‐C

In a total of 4724 patients in 46 RCTs,^17^, ^18^, ^19^, ^20^, ^21^, ^22^, ^23^, ^24^, ^25^, ^26^, ^27^, ^28^, ^29^, ^30^, ^31^, ^32^, ^33^, ^34^, ^35^, ^36^, ^37^, ^38^, ^39^, ^40^, ^41^, ^42^, ^43^, ^44^, ^45^, ^46^, ^47^, ^48^, ^49^, ^50^, ^51^, ^52^, ^53^, ^54^, ^55^, ^56^, ^57^, ^58^, ^59^, ^60^, ^61^, ^62^ three types of Chinese patent medicines were analysed for changes in LDL‐C levels compared to Simvastatin or placebo. Chi‐squared tests showed no heterogeneity between studies (*p* = 1.0, *I* ^2^ = 0.0%). There was a statistically significant difference in all treatments compared with the placebo group. No statistically significant differences were observed between the other comparisons. The results of the network meta‐analysis are summarized in Table 4. The ranking probabilities based on SUCRA values (Figure 6) were Xuezhikang (82.6%) > Simvastatin (74.9%) > Zhibituo (52.8%) > Zhibitai (39.5%) > placebo (0.2%), indicating that Xuezhikang was the most effective intervention for LDL‐C, followed by Simvastatin and Zhibituo, respectively.

**TABLE 4:** Network meta‐analysis for LDL‐C (MD [95% CI])

Zhibituo				
−0.16 (−0.68, 0.36)	Zhibitai			
0.12 (−0.14, 0.38)	0.28 (−0.23, 0.79)	Xuezhikang		
0.09 (−0.12, 0.30)	0.25 (−0.23, 0.72)	−0.03 (−0.21, 0.15)	Simvastatin	
**−0.94 (−1.35**, **−0.52)**	**−0.78 (−1.38**, **−0.18)**	**−1.06 (−1.38**, **−0.73)**	**−1.02 (−1.39**, **−0.65)**	Placebo

Bold values indicate statistically significant values *p* <  0.05.

#### 3.4.5 HDL‐C—High‐density lipoprotein cholesterol levels

**FIGURE 7:**
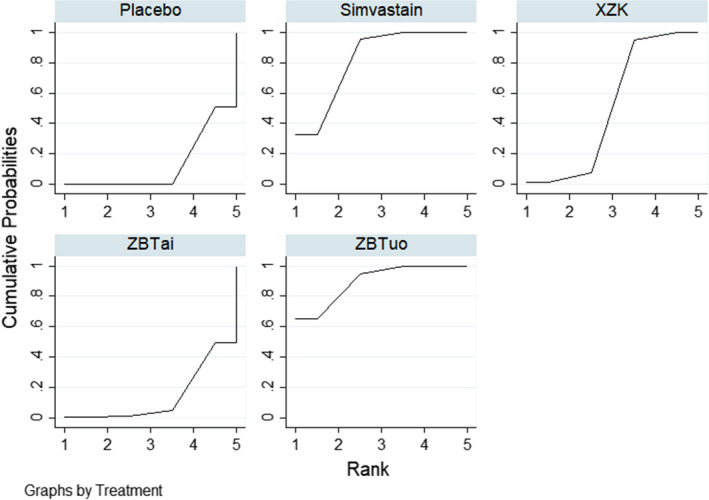
SUCRA curves for HDL‐C

A total of 4473 patients in 43 RCTs^17^, ^18^, ^19^, ^20^, ^21^, ^22^, ^23^, ^24^, ^25^, ^26^, ^27^, ^28^, ^29^, ^32^, ^34^, ^35^, ^36^, ^38^, ^39^, ^40^, ^41^, ^42^, ^43^, ^44^, ^45^, ^46^, ^47^, ^48^, ^49^, ^50^, ^51^, ^52^, ^53^, ^54^, ^55^, ^56^, ^57^, ^58^, ^59^, ^60^, ^61^, ^62^, ^63^ were used to analyse three Chinese patent medicines for changes in HDL‐C levels compared to Simvastatin or placebo. Heterogeneity tests showed no heterogeneity between studies (*p* = .24, *I* ^2^ = 0.0%). One study^30^ was excluded from the analysis because it identified an outlier value. The results suggested that in five comparisons (Zhibituo vs. Zhibitai; Zhibituo vs. placebo; Simvastatin vs. Zhibitai; Xuezhikang vs. placebo; Simvastatin vs. p‐placebo), the differences were statistically significant (MD = 0.16, 95% CI [0.02, 0.30]; MD = 0.16, 95% CI [0.07, 0.25]; MD = −0.15, 95% CI [−0.28, −0.02]; MD = 0.11, 95% CI [0.03, 0.18]; MD = 0.1, 95% CI [0.06, 0.23], respectively). No statistically significant differences were observed between the other comparisons. The results of the network meta‐analysis are summarized in Table 5. The ranking probabilities based on SUCRA values (Figure 7) were as follows: Zhibituo (90.1%) > Simvastatin (82.1%) > Xuezhikang (51.1%)> Zhibitai (14.0%) > placebo (12.7%), indicating that Zhibituo was the most effective intervention for HDL‐C, followed by Simvastatin and Xuezhikang.

**TABLE 5:** Network Meta‐analysis of HDL‐C (MD [95% CI])

Zhibituo				
0.16 **(0.02**, **0.30)**	Zhibitai			
0.05 (−0.01, 0.11)	−0.11 (−0.24, 0.03)	Xuezhikang		
0.01 (−0.04, 0.06)	−0.15 **(**−0.28, −0.02**)**	−0.04 (−0.09, 0.00)	Simvastatin	
**0.16 (0.07**, **0.25)**	−0.00 (−0.15, 0.15)	**0.11 (0.03**, **0.18)**	**0.15 (0.06**, **0.23)**	Placebo

Bold values indicate statistically significant values *p* <  0.05.

### 3.5 Adverse drug reactions

No serious adverse events were reported in the 47 RCTs included in this study. Of the 40 trials that described adverse reactions during treatment, 13 reported no adverse reactions, while 27 RCTs reported adverse events in detail.

Of the interventions that involved treatment with Simvastatin, 26 RCTs (1546 patients in total) reported adverse events, including gastrointestinal symptoms (80 cases, such as abdominal pain, bloating and nausea), slightly increased aspartate aminotransferase (AST) and alanine aminotransferase (ALT) levels (41 cases), headache (1 case), fatigue (4 cases) and muscle spasms (3 cases).

For treatment with Zhibituo, 15 RCTs (a total of 837 patients) reported adverse events, including gastrointestinal symptoms (35 cases, such as abdominal pain, bloating and nausea).

Thirteen RCTs (a total of 811 patients) reported adverse events after treatment with Xuezhikang, including gastrointestinal symptoms (39 cases, such as abdominal pain, bloating and nausea) and slightly increased levels of AST and ALT (6 cases).

### 3.6 Publication bias

**FIGURE 8:**
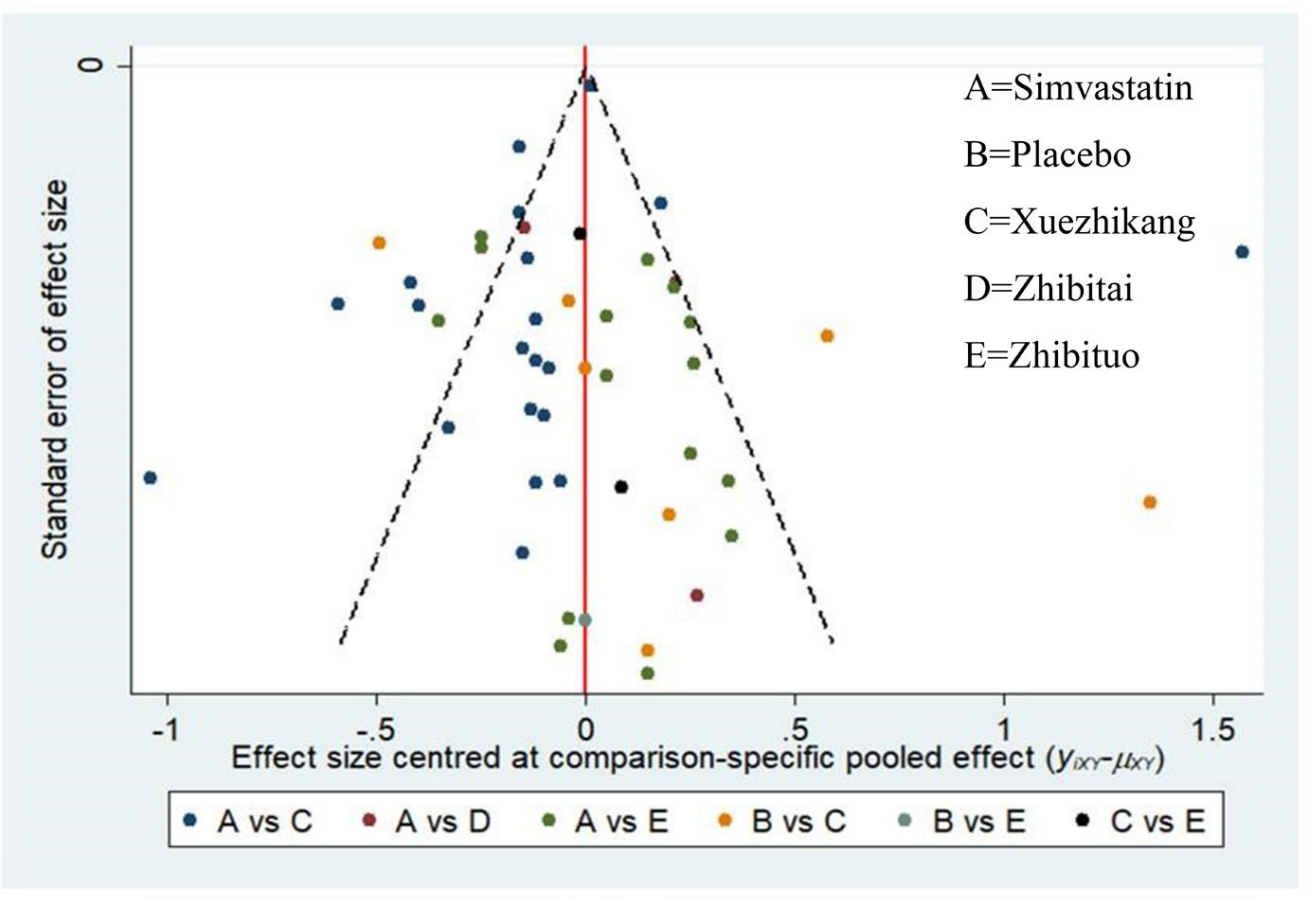
Funnel plot for TC. A Simvastatin; B Placebo; C Xuezhikang; D Zhibitai, E Zhibituo

A comparison‐adjusted funnel plot of all outcomes demonstrated, by its asymmetry, that some publication bias existed, for which that of TC is displayed in Figure 8.

### 3.7 Quality of evidence

The GRADE approach was used to assess the quality of evidence. The quality of evidence for the outcomes was low, and the results are presented in Table 6, and the reasons for downgrading included study limitations (risk of bias) and imprecision. Most of the included studies were classified as high risk; there was imprecision because the ranking probabilities based on SUCRA values were very close, and publication bias was observed.

**TABLE 6:** Results of GRADE evidence evaluation

Outcome	No. of participants (studies)	Certainty of evidence	Downgrading due to
TC	4803 (47)	Low	Study limitations; publication bias
TG	4591 (45)	Low	Study limitations; publication bias
LDL‐C	4724 (46)	Very low	Study limitations; imprecision; publication bias
HDL‐C	4473 (43)	Very low	Study limitations; imprecision; publication bias

## 4 DISCUSSION

The incidence of hyperlipidaemia has increased owing to heredity, nutrition, diet, medication and other factors.^64^ Hyperlipidaemia is a major risk factor for cardiovascular diseases and atherosclerosis.^65^ There is increasing evidence that traditional Chinese medicines that eliminate phlegm and blood stasis can successfully reverse the symptoms of hyperlipidemia.^66^, ^67^ Previous meta‐analyses have compared the efficacy and safety of RYR for hyperlipidaemia. In 2006, Liu et al.^68^ compared the effectiveness of RYR with placebo, no treatment, statins or other active lipid‐lowering agents in the treatment of hyperlipidaemia, whereas the control group in our study received Simvastatin (Zocor); the inclusion criteria in our study were more specific. In 2014, only 13 RCTs were included by Li et al.,^69^ and in 2015, 20 studies were included by Gerards et al.^70^ However, 47 RCTs were included in our study. In 2019, Fogacci et al.^71^ performed a meta‐analysis on the safety data surrounding RYR, whereas the purpose of our study was to evaluate the therapeutic effectiveness. In 2020, Sungthong et al.^72^ performed a meta‐analysis to analyse the efficacy of RYR on cardiovascular outcomes in patients with myocardial infarction, while the participants of our study were diagnosed with hyperlipidaemia. RYR showed overall tolerability and safety for hyperlipidaemia, based on a previous meta‐analysis.^71^ The results of our meta‐analysis provide evidence that Chinese patent medicines containing RYR are highly efficient for the treatment of hyperlipidaemia.

The present network meta‐analysis is the first study to assess and rank the effectiveness of Chinese patent medicines that eliminate phlegm and remove blood stasis in treating hyperlipidaemia. By adopting rigorous inclusion criteria, 47 RCTs with 4824 participants were included in the analyses. The results indicated that different Chinese patent medicines have different benefits for the treatment of hyperlipidaemia. In terms of reducing the levels of TC, Xuezhikang (SUCRA: 84.5%) displayed the highest probability of being the most effective option, followed by Simvastatin (SUCRA: 66.4%) and Zhibitai (SUCRA: 65.4%). For reducing TG levels, Zhibituo (SUCRA: 80.2%) exhibited the highest probability of being the most effective, with Xuezhikang (SUCRA: 63.4%) and Simvastatin (SUCRA: 48.7%) in second and third places, respectively. In terms of reducing LDL‐C levels, Xuezhikang (SUCRA: 82.6%) had the highest probability of being the most effective, followed by Simvastatin (SUCRA: 74.9%) and Zhibituo (SUCRA: 52.8%). Finally, in terms of improving HDL‐C levels, Zhibituo (SUCRA: 90.1%) had the highest probability of being the most effective, with Simvastatin (SUCRA: 82.1%) and Xuezhikang (SUCRA: 51.1%) ranked second and third, respectively. Furthermore, no additional severe toxicity was identified in any experimental group compared to the control group. However, there was no significant difference between Zhibituo and Xuezhikang.

The RYR has been widely used in China for many years.^73^ Previous studies have shown that it can reverse the symptoms of hyperlipidaemia, the mechanism of action of which is similar to that of statins.^74^ Statins are the key lipid‐lowering medications and are the current recommended initial therapy for blood lipid disorders.^75^, ^76^ The mechanism is that its efficacy component, monacolin K, acts like the synthetic drug Lovastatin but without the severe side effects of statins.^77^ In addition, experimental studies have indicated that the main chemical component of RYR, responsible for its lipid‐reducing properties, is ergosterol.^78^ Clinical studies have suggested that other compounds in RYR may also decrease serum lipid levels.^79^ Our network meta‐analysis yielded results similar to those of previous studies.

Both Xuezhikang and Zhibituo are made from RYR, which can alleviate drug properties, enhance or change drug effects, reduce toxicity and expand the range of clinical applications of fermented medicines.^80^ However, there are different active ingredients in Xuezhikang and Zhibituo.^81^ Xuezhikang is made by high‐tech biotechnology, containing Lovastatin, a statin homolog, a variety of essential amino acids, unsaturated fatty acids, sterols and small amounts of flavonoids.^82^, ^83^ The main bioactive components of Zhibituo are Lovastatin and Lovastatin acid.^84^ The content of Lovastatin in Zhibituo and Xuezhikang was shown to be 2.7 and 11.1 g/kg, respectively.^81^ Xuezhikang provides hypotriglyceridemic performance superior to Simvastatin in terms of reduction in levels, and the underlying mechanism has been attributed to more significant apoA5 upregulation via the PPARα signalling pathway.^85^ Proprotein convertase subtilisin/kexin type 9 (PCSK9) is a crucial regulator of plasma cholesterol homeostasis.^86^ A previous study demonstrated that Xuezhikang increases PCSK9 levels through the SREBP‐2 pathway,^87^ and isoflavones and phytosterols in Xuezhikang play a role in lowering cholesterol levels through a mechanism different from that of Lovastatin, which elevates the excretion of lipids and bile acids in faeces.^88^ In summary, Xuezhikang and Zhibituo contain natural statins, which are safer than synthetic statins and can lower blood lipids.^89^

However, the present study has several limitations: (1) all studies included in the review were from China, which might be a potential source of publication bias. (2) Publication bias was also observed. (3) The quality of the studies included in the review was not considered high, with five studies^21^, ^22^, ^28^, ^37^, ^46^ reporting the specific methods of random sequence generation, three randomized, double‐blind, placebo‐controlled trials^36^, ^52^, ^53^ and four randomized, placebo‐controlled trials.^50^, ^58^, ^61^, ^63^ In addition, the quality of evidence for the outcomes was low. (4) The number of trials that compared some of the medicines was relatively small, causing the comparative results to not be incredibly persuasive, and those cases should be considered with caution. Thus, additional, well‐designed, double‐blinded, multicentre RCTs are required to establish the efficacy of Chinese patent medicines for the treatment of hyperlipidaemia.

## 5 CONCLUSIONS

To reduce the levels of TC and LDL‐C, Xuezhikang displayed the highest probability of being the most effective option. To reduce TG levels, Zhibituo exhibited the highest probability of being the most effective, and Zhibituo may have the highest probability of ameliorating levels of HDL‐C, whereas there was no significant difference between Zhibituo and Xuezhikang. Owing to the limitations of this study, the validity of our results requires confirmation using large‐sample, high‐quality, multicentre, prospective RCTs.

## CONFLICT OF INTEREST

The authors declare that they have no conflicts of interest regarding this study.

## AUTHOR CONTRIBUTIONS

**Guiqin Xu:** Conceptualization (equal); Data curation (equal); Software (equal); Writing‐original draft (lead); Writing‐review & editing (equal). **Mingxin Lin:** Conceptualization (equal); Data curation (equal); Project administration (lead); Software (lead); Writing‐original draft (lead); Writing‐review & editing (lead). **Xueli Dai:** Data curation (equal); Software (equal). **Jingqing Hu:** Conceptualization (lead); Methodology (lead); Writing‐original draft (equal); Writing‐review & editing (equal).

## Data Availability

The data that support the findings of this study are available from the corresponding author upon reasonable request.
